# New Broth Macrodilution Volatilization Method for Antibacterial Susceptibility Testing of Volatile Agents and Evaluation of Their Toxicity Using Modified MTT Assay In Vitro

**DOI:** 10.3390/molecules26144179

**Published:** 2021-07-09

**Authors:** Marketa Houdkova, Aishwarya Chaure, Ivo Doskocil, Jaroslav Havlik, Ladislav Kokoska

**Affiliations:** 1 Department of Crop Sciences and Agroforestry, Faculty of Tropical AgriSciences, Czech University of Life Sciences Prague, 16500 Prague, Czech Republic; houdkovam@ftz.czu.cz (M.H.); chaure@ftz.czu.cz (A.C.); 2 Department of Microbiology, Nutrition and Dietetics, Faculty of Agrobiology, Food and Natural Resources, Czech University of Life Sciences Prague, 16500 Prague, Czech Republic; doskocil@af.czu.cz; 3 Department of Food Science, Faculty of Agrobiology, Food and Natural Resources, Czech University of Life Sciences Prague, 16500 Prague, Czech Republic; havlik@af.czu.cz

**Keywords:** antimicrobial, cytotoxicity, macrodilution method, respiratory infections, β-thujaplicin, thymohydroquinone, thymoquinone, vapor phase, volatile compound

## Abstract

In this study, a new broth macrodilution volatilization method for the simple and rapid determination of the antibacterial effect of volatile agents simultaneously in the liquid and vapor phase was designed with the aim to assess their therapeutic potential for the development of new inhalation preparations. The antibacterial activity of plant volatiles (β-thujaplicin, thymohydroquinone, thymoquinone) was evaluated against bacteria associated with respiratory infections (*Haemophilus influenzae*, *Staphylococcus aureus*, *Streptococcus pneumoniae*, *Streptococcus pyogenes*) and their cytotoxicity was determined using a modified thiazolyl blue tetrazolium bromide assay against normal lung fibroblasts. Thymohydroquinone and thymoquinone possessed the highest antibacterial activity against *H. influenzae*, with minimum inhibitory concentrations of 4 and 8 µg/mL in the liquid and vapor phases, respectively. Although all compounds exhibited cytotoxic effects on lung cells, therapeutic indices (TIs) suggested their potential use in the treatment of respiratory infections, which was especially evident for thymohydroquinone (TI > 34.13). The results demonstrate the applicability of the broth macrodilution volatilization assay, which combines the principles of broth microdilution volatilization and standard broth macrodilution methods. This assay enables rapid, simple, cost- and labor-effective screening of volatile compounds and overcomes the limitations of assays currently used for screening of antimicrobial activity in the vapor phase.

## 1. Introduction

Bacterial infections of the lower respiratory tract, such as pneumonia and bronchitis, are some of the leading global causes of death, especially among children under five years of age and elderly people [1,2]. Typical causative bacterial species of respiratory infections include *Haemophilus influenzae*, *Staphylococcus aureus*, *Streptococcus pneumoniae*, and *Streptococcus pyogenes* [3]. Moreover, co-infection with viruses (e.g., severe acute respiratory syndrome coronavirus) can significantly increase the morbidity and mortality rates [4]. Inhalation therapy represents the appropriate way to treat respiratory disorders. If a medication is inhaled, it is directly delivered into the airways to the site of infection. This gives a relatively faster onset of action, while using a lower dose of active agent, which consequently causes fewer side effects in districts where its action is not needed [5,6]. Although antibiotic treatment is generally considered as effective against most infective strains, it is increasingly failing due to some limitations including allergies, bacterial resistance, inadequate penetration in lung tissues, and undesirable adverse effects [7]. Several types of devices for the delivery of inhaled medications to the lungs have been invented, such as nebulizers, pressurized metered-dose and dry-powder inhalers; however, they all face various limitations, including a short-life because of pulmonary clearance, enzymatic degradation, fast systemic absorption, and poor bioavailability of bioactive agents at the target site [8]. Moreover, the efficiency of the inhalable therapies may be affected by the deposition of the aerolized particles in the oropharyngeal region and upper airways while the deposition of drugs in the lungs can be reduced due to the inappropriate size of its droplets or due to specific respiratory tract anatomy, and proper operation, especially in children and elderly patients [9,10].

Since plant-derived antimicrobial agents have been traditionally used in the prevention and treatment of respiratory infections, they are considered promising sources of novel chemical scaffolds for the development of new drugs against bacteria causing respiratory diseases [11]. For this reason, the biological potential of natural substances, including volatile plant-derived compounds has been intensively studied in recent years with the aim to find potential alternatives to conventional synthetic antimicrobial agents [12,13]. For example, β-thujaplicin, a monoterpenoid isolated from the wood of the Cupressaceae species, and thymohydroquinone, a monoterpenoid phenol occurring in the Ranunculaceae and Lamiaceae families, have been reported to exhibit antimicrobial properties, including effects against bacteria causing respiratory infections [14,15,16,17]. Due to the high vapor pressure of volatile plant-derived products at the ambient temperature, they have the benefit of bioactivity in the vapor phase [18], and thus, plant volatiles have great potential for the development of novel preparations for inhalation [19,20]. For example, thymoquinone, a benzoquinone occurring in the seeds of *Nigella sativa* (Ranunculaceae), has been recorded to possess a relatively strong antibacterial effect in the vapor phase against pathogens that cause pneumonia [21]. Although plant-derived products are generally considered as relatively safe, the toxicological evaluation of volatile agents is necessary to confirm their non-toxicity for their practical application in inhalation therapy [22]. Despite the availability of several lung cell in vitro models, there is an urgent need for the development of more appropriate non-animal methods of inhalation toxicity, particularly for predicting effects in humans [23].

In vitro screening is typically the first step in the process of discovery of new antimicrobial drugs, including those derived from plant volatiles. However, the susceptibility testing of microorganisms to volatile agents using standard methods, such as broth dilution and disk diffusion assays, is a challenging task because of their specific physico-chemical properties, including high volatility, hydrophobicity, and viscosity [24]. The main problem is that their hydrophobic nature worsens the solubility of these compounds in water-based media (e.g., agar, broth) and their volatility increases the risk of loss of active substances via evaporation during sample handling, experiment preparation, and incubation. In addition, the transition of the vapors can affect the microplate assay results, as described in our previous studies [25,26]. This is even more complicated in the case of antimicrobial vapors testing. In contrast to well-established methods for antimicrobial susceptibility testing on solid (agar disc diffusion) and liquid (broth dilution) media [27,28,29,30,31,32], there are no standardized methods for the determination of microbial sensitivity to volatile compounds in the vapor phase. In recent years, several methods for the testing of the antimicrobial effects of volatile plant-derived products in the vapor phase have been developed. However, most of them have some specific limitations, such not being designed for high-throughput screening, and some of them need special equipment that is not commonly available [33]. Recently, we proposed a broth microdilution volatilization assay [21] based on the principles of broth microdilution and disc volatilization methods, which is suitable for high-throughput screening of volatile compounds simultaneously in the liquid and vapor phase. This method can also be easily used for determination of the antibacterial effects of essential oil vapors [34,35]. Although the broth microdilution volatilization method is fast, simple and labor-effective, it has several weaknesses. For example, clamps and wooden pads are required for a better sealing and fixing the microtiter plate and its lid together. Moreover, the limited volume of agar that is applied on the lid can affect the growth of the microorganisms tested.

**Figure 1:**
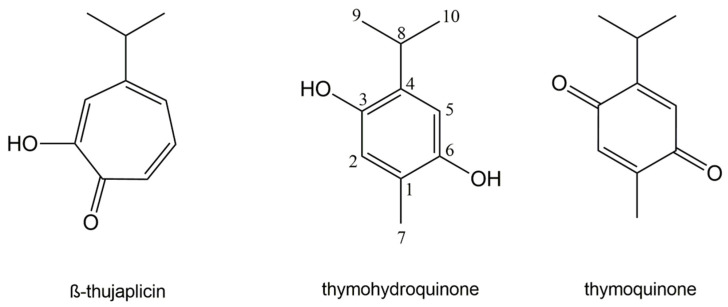
Chemical structures of plant volatile compounds tested.

With the aim to overcome the above mentioned drawbacks of previously developed methods used for testing of volatile antimicrobial agents in the vapor phase, we designed a novel macrodilution volatilization assay that combines the principles of broth microdilution volatilization [21] and standard broth macrodilution [27] methods. The validity of the method for susceptibility testing of bacterial pathogens causing respiratory infections was evaluated using three antimicrobial phytochemicals, namely β-thujaplicin, thymohydroquinone, and thymoquinone (Figure 1). In addition, the cytotoxicity of these compounds was analyzed using a modified thiazolyl blue tetrazolium bromide (MTT) cytotoxicity assay to assess their safety for use in the treatment of respiratory infections.

## 2. Results and Discussion

### 2.1. Antimicrobial Activity

The results of the antibacterial effect of plant-derived volatiles against respiratory pathogens in the liquid and vapor phases assessed using the broth macrodilution volatilization method are listed in Table 1. All the compounds tested exhibited a certain degree of growth-inhibitory effect in both the liquid and vapor phase and their effectiveness varied in the ranges 4–64 µg/mL and 8–1024 µg/mL in the broth and agar media, respectively. To the best of our knowledge, this is the first report on the antibacterial activity of thymohydroquinone in the vapor phase. Moreover, the antimicrobial susceptibility of *H. influenzae* to β-thujaplicin and thymohydroquinone, *S. pyogenes* to thymohydroquinone and thymoquinone, and *S. pneumoniae* to thymohydroquinone was also described for the first time in this study.

In the liquid phase, the lowest minimal inhibitory concentration (MIC) values were observed for thymohydroquinone and thymoquinone against *H. influenzae* (4 µg/mL), followed by the growth-inhibitory effects against *S. aureus*, *S. pneumoniae*, and *S. pyogenes* with respective MIC values of 8, 16, and 32 µg/mL. β-thujaplicin exhibited the same level of antibacterial activity against all bacterial strains tested with a MIC of 64 µg/mL. As well as in the broth, thymohydroquinone and thymoquinone were found to be the most active antibacterial agents against *H. influenzae* in the vapor phase with a MIC of 8 µg/mL. In addition, both compounds effectively inhibited the growth of *S. aureus* and *S. pyogenes* on agar medium at the same concentrations, 16 and 32 µg/mL, respectively. However, their activity differed against *S. pneumoniae*, where a lower MIC value was detected for thymoquinone (16 µg/mL) than for thymohydroquinone (32 µg/mL). In the case of β-thujaplicin, a moderate antibacterial efficacy was recorded against *H. influenzae* and *S. aureus* (512 µg/mL) and a low antibacterial efficacy against *S. pneumoniae* and *S. pyogenes*.

In general, the results of antibacterial potential of plant-derived volatiles obtained by our novel broth macrodilution volatilization assay correspond with those observed by other authors using standard broth and agar dilution methods. The MICs previously determined by Domon et al., Morita et al., and Inoue et al. [17,36,37] for β-thujaplicin against various strains of *S. aureus* in the range 12.5–160 µg/mL were close to the MIC detected in our study. However, a much higher antimicrobial efficacy of this compound was recorded against *S. pneumoniae* and *S. pyogenes* with MICs of 0.3–1 µg/mL in previously published studies [17]. Other authors [25,38] observed the growth-inhibitory effect of thymoquinone against *S. aureus* ATCC 29213 and ATCC 25923 with the MIC value of 8 µg/mL, which is the same as that in our study. In the case of thymohydroquinone, a fifty-times-higher MIC value of 400 µg/mL was detected against *S. aureus* ATCC 25923 [39]. As it has previously been observed, various bacterial strains with different susceptibilities to antibacterial agents [40], as well as different antimicrobial assays used for the testing of volatiles [41], may be responsible for the variability of the results obtained by other authors. In contrast to a number of published papers on the antibacterial potential of plant-derived volatile compounds in the liquid phase, literature on the activity of their vapors is limited. Wang et al. [42] recorded a certain level of growth-inhibitory effect of β-thujaplicin against oral microorganisms in the gaseous phase and Inouye et al. [43] described a very potent vapor activity of thymoquinone against *Trichophyton mentagrophytes*; both by using the disc volatilization method. Our previous results on the antimicrobial activity of thymoquinone against respiratory pathogens *H. influenzae*, *S. aureus*, and *S. pneumoniae* obtained by using the broth microdilution volatilization assay with respective MIC values of 8, 16, and 16 µg/mL in the liquid phase and 8, 16, and 32 µg/mL in the vapor phase [21] correspond to this current study and demonstrate the validity of our newly developed method. In general, the compounds tested in this study showed higher or equal antibacterial effects in the liquid phase than in the vapor phase. The largest differences were observed in the case of β-thujaplicin when sixteen times lower MIC values were detected in broth than on the agar against *S. pneumonieae* and *S. pyogenes*.

The above-described results demonstrate the validity of our novel broth macrodilution volatilization assay, which combines the principles of broth microdilution volatilization [21] and standard macrodilution methods [27] and overcomes some of their drawbacks. In comparison with previously established liquid matrix volatilization techniques, the broth macrodilution volatilization method is performed in commercially available microtubes, which can be tightly closed with a snap cap, and therefore, it does not require specialized equipment, such as wooden pads and clamps to prevent the losses of the active agents by evaporation. A variable number of sample concentrations tested in one experiment is another benefit of this method, while the design is not limited by the quantity of wells, as in the case of microplate-based assays. Whereas a higher amount of appropriate media can be applied in microtubes and their caps, which are suitable for the cultivation of slower growing microorganisms (e.g., fungi) that require longer incubation time to produce enough growth for MIC determination [44]. Despite the obvious benefits of our novel antimicrobial susceptibility test based on microtubes, it has some specific limits, such as a lower potential for the automation (e.g., use of automated pipetting platform and reader) and the need for an extra step for the preparation of an appropriate amount of serially diluted concentrations of samples in the test tubes. Due to the transition of antimicrobial compounds between the liquid and vapor phases and their possible losses during the experiment preparation, the final concentration should be considered as indicative only. For that reason, the concentrations of the samples tested in the vapor phase were expressed as the weight of the volatile agent per volume unit of a microtube, that is, 640, 320, 160, 80, 40, 20, 10, and 5 µg/cm^3^ for 1024, 512, 128. 64, 32, 16, 8, and 4 µg/mL, respectively. Nevertheless, the real quantity of volatile agents evaporated from the broth should be determined, e.g., using a combination of solid-phase microextraction and gas chromatography analysis [45].

### 2.2. Cytotoxicity

**Figure 2:**
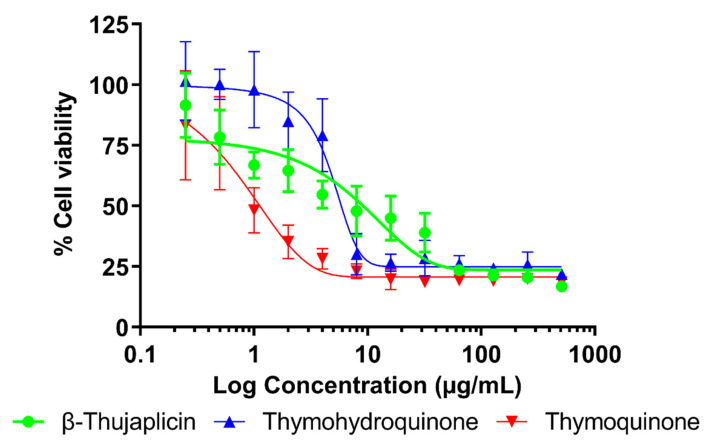
Cytotoxic activity of twelve two-fold serially diluted concentrations (0.25–512 µg/mL) of plant volatile compounds to lung fibroblast cells tested by using MTT assay performed in microtiter plates sealed with vapor barrier EVA Capmat.

The results of the modified MTT assay performed on lung fibroblast cells are summarized in Table 2. The cytotoxic effect of twelve two-fold serially diluted concentrations of compounds tested are displayed in Figure 2. *β*-Thujaplicin and thymohydroquinone exhibited moderate toxicity to the lung cells with respective half maximal inhibitory concentration (IC_50_) values of 4.15 and 2.64 µg/mL. Thymoquinone was evaluated as toxic with an IC_50_ value of 1.21 µg/mL. In the case of an 80% inhibitory concentration of proliferation (IC_80_) determination, the lowest cytotoxic effect was observed for thymohydroquinone followed by β-thujaplicin with IC_80_ values >12.00 and 214.85 µg/mL, respectively. Similar to IC_50_, the lowest IC_80_ value was recorded for thymoquinone (IC_80_ = 15.00 µg/mL). Although according to the WHO [46], all the compounds were classified as toxic and moderately toxic, the therapeutic index (TI) calculated to compare their antibacterial and cytotoxic effects indicate that thymohydroquinone (TI > 34.13) can be a safe and effective antibacterial agent for inhalation therapy.

Series of assays examining the toxic potential of thymoquinone have been performed including our previous study [21], which observed that this compound has a very similar cytotoxic effect on healthy human lung MRC-5 cell lines with a IC_50_ value of 1.70 µg/mL, while Gurung et al. [47] found that thymoquinone did not alter the viability of normal IMR-90 lung fibroblasts at a concentration of 32.84 µg/mL. In contrast to the well-documented toxicity of thymoquinone, there are only few data available on the safety of other compounds tested in normal human lung fibroblasts. In a study of Ivankovic et al. [48], thymohydroquinone exhibited a certain level of cell growth inhibition against mouse fibroblasts at concentrations of 10 and 100 µg/mL (21% and 63%, respectively). A toxic effect of β-thujaplicin was detected against various types of human lung cancer tissues, with IC_50_ values of 0.26–12.32 µg/mL [49,50,51]. Regarding the relatively high toxicity of compounds tested in lung cell cultures, their practical application for inhalation therapy to treat respiratory infection seems to be limited. However, their specific structural and technological modifications reducing toxicity are possible. For example, liposomal encapsulation of thymoquinone was found to be effective in decreasing its toxic effects [52].

## 3. Materials and Methods

### 3.1. Chemicals and Reagents

The following plant-derived volatile antibacterial agents were assayed: β-thujaplicin (99%, CAS 499-44-5), thymohydroquinone (98%, CAS 2217-60-9), and thymoquinone (99%, CAS 490-91-5). Amoxicillin (90%, CAS 26787-78-0), ampicillin (84.5%, CAS 69-52-3), oxacillin (86.3%, CAS 7240-38-2), and tetracycline (98–102%, CAS 60-54-8) were used as positive antibiotic controls. The chemicals used for antimicrobial susceptibility testing were as follows: dimethyl sulfoxide (DMSO, CAS 67-68-5), thiazolyl blue tetrazolium bromide dye (MTT, CAS 298-93-1), and Tween 20 (CAS 9005-64-5). With the exception of thymohydroquinone prepared by reduction of thymoquinone according to a method described further, all other chemicals were obtained from Sigma-Aldrich (Prague, Czech Republic).

The following chemicals were used for thymohydroquinone preparation and characterization: acetic acid (CAS 64-19-7) purchased from Merck (Darmstadt, Germany), then deuterium oxide containing 3-(trimethylsilyl) propionic-2,2,3,3-*d_4_* acid sodium salt (CAS 7789-20-0), vanillin (CAS 121-33-5) and zinc (CAS 7440-66-6) purchased from Sigma-Aldrich (Praha, Czech Republic), and others obtained from Penta (Praha, Czech Republic), namely chloroform (CAS 67-66-3), ethanol (96%, CAS 64-17-5), hexane (CAS 110-54-3), hydrochloric acid (35%, CAS 7647-01-0), and sulfuric acid (CAS 7664-93-9).

### 3.2. Thymohydroquinone Preparation and Characterization

Thymohydroquinone was prepared by the reduction of thymoquinone using acetic acid in the presence of zinc powder as a catalyst according to the method previously described by Tesarova et al. [53]. Briefly, 50 mg of thymoquinone (Sigma Aldrich, Praha, Czech Republic) was dissolved in 3 mL of concentrated acetic acid (99%) and 5 g of zinc powder was subsequently added. The reaction mixture was stirred for 4 h, monitored by thin layer chromatography (TLC) under laboratory conditions, and then filtered. The TLC was performed on TLC Silica gel 60 F254 (Merck, Darmstadt, Germany) plates with chloroform and ethanol in a ratio of 9:1 as the mobile phase. Vanillin dissolved in sulfuric acid in ethanol (1%) was used as a visualizing agent. The liquid fraction was evaporated under vacuum in a rotary evaporator (Rotavapor R-210, Buchi, Flawil, Switzerland). The solid residue was dissolved in water, acidified by the addition of hydrochloric acid (to 5% *v/v*) and yellow residues of thymoquinone were removed by repeated extraction with hexane. Thymohydroquinone was then extracted three times with distilled diethyl ether and evaporated under a stream of N_2_. For further purification, sublimation at 168 °C was used and colorless needle crystals of thymohydroquinone were stored in the dark.

The identity and purity of obtained thymohydroquinone were confirmed using gas chromatography/mass spectrometry analysis (GC/MS) and a nuclear magnetic resonance (NMR). The mass spectra of thymohydroquinone were recorded by Agilent GC-7890B and MSD-5977B (Agilent Technologies, Santa Clara, CA, USA) equipped with a fused-silica HP-5MS column (30 m × 0.25 mm, film thickness 0.25 μm, Agilent 19091s-433) and a flame ionization detector coupled with single quadrupole mass selective detector Agilent MSD-5977B (Agilent Technologies, Santa Clara, CA, USA). Operational parameters were: helium as a carrier gas at 1 mL/min, injector temperature 250 °C. The oven temperature was raised from 50 to 280 °C. Thymohydroquinone was diluted in *n*-hexane for GC/MS at a concentration of 20 µg/mL. One microliter of solution was injected in a split mode (split ratio 1:50). The mass detector was set to the following conditions: ionization energy 70 eV, ion source temperature 230 °C, scan time 1 s, mass range 40–600 *m*/*z*. Identification of the sample was based on the comparison of its retention index (RI), retention time (RT) and mass spectra with the National Institute of Standards and Technology Library ver. 2.0.f (National Institute of Standards and Technology, USA) [54].

The ^1^H-NMR spectra of thymohydroquinone standard (2 mg/mL) were recorded on a Bruker Avance III HD BBFO (Bruker BioSpin GmbH, Rheinstetten, Germany), operating at 500 MHz for ^1^H-NMR and 126 MHz for ^13^C-NMR using *noesypr1d* pulse sequence, at 25 °C. The sample was dissolved in H_2_O containing 10% deuterium oxide, at pH 7.4, and including 3-(trimethylsilyl) propionic-2,2,3,3-*d_4_* acid sodium salt (99%) as an internal standard. Supplementary evidence was given by ^13^C-NMR, HSQC, HMBC and COSY experiments. The experimental chemical shifts in ^1^H and ^13^C-NMR spectra of thymohydroquinone closely matched the theoretically predicted chemical shifts obtained using www.nmrdb.org (accessed on 21 June 2021) [55,56].

The signals were confirmed and assigned after inspection of the 1D and 2D spectra and GC/MS data and were as follows: *Thymohydroquinone*. White crystals; RI: 1520, MS, *m*/*z* (rel. int.): 166 (M^+^, 43%), 151 (100), 152 (14), 77 (8), 123 (7), 95 (7); ^1^H-NMR (H_2_O/D_2_O 9:1, 500 MHz): *δ* 6.79 (s, 1H, 2-H), 6.73 (s, 1H, 5-H), 3.14 (sept, 1H, *J* = 6.9 Hz, 8-H), 2.13 (s, 3H, 7-H), 1.17 (d, 6H, *J* = 6.9 Hz, 9,10-H); ^13^C-NMR (H_2_O/D_2_O 9:1, 126 MHz): *δ* 147.5 (C-6), 145.8 (C-3), 134.4 (C-4), 123.3 (C-1), 118.3 (C-2), 113.2 (C-5), 26.0 (C-8), 22.2 (C-9), 22.2 (C-10), 14.9 (C-7).

### 3.3. Bacterial Strains and Culture Media

The following four bacterial standard strains from the American Type Culture Collection (ATCC, Manassas, VA, USA) were used: *Haemophilus influenzae* ATCC 49247, *Staphylococcus aureus* ATCC 29213, *Streptococcus pneumoniae* ATCC 49619, and *Streptococcus pyogenes* ATCC 19615. Cultivation and assay media (broth/agar) were Mueller–Hinton (MH) complemented by Haemophilus Tested Medium (*H. influenzae*), MH (*S. aureus*), and Brain Heart Infusion (*S. pneumoniae* and *S. pyogenes*). The pH of the broths was equilibrated to a final value of 7.6 using Trizma base (Sigma-Aldrich, Praha, Czech Republic). All microbial strains and cultivation media were purchased from Oxoid (Basingstoke, UK).

Stock cultures of bacterial strains were cultivated in broth medium at 37 °C for 24 h prior to testing. For the preparation of inoculum, the turbidity of the bacterial suspension was adjusted to 0.5 McFarland standard using a Densi-La-Meter II (Lachema, Brno, Czech Republic) to obtain a final concentration of 10^8^ CFU/mL.

### 3.4. Cell Cultures

Lung fibroblast cell line MRC-5, obtained from ATCC, was propagated in Eagle’s Minimum Essential Medium (EMEM) supplemented with 10% fetal bovine serum (FBS), 2 mM glutamine, 10 µL/mL non-essential amino acids, and 1% penicillin–streptomycin solution (10,000 units/mL of penicillin and 10 mg/mL of streptomycin); all these components were purchased from Sigma-Aldrich. The cells were pre-incubated in 96-well microtiter plates at a density of 2.5 × 10^3^ cells per well for 24 h at 37 °C in a humidified incubator in an atmosphere of 5% CO_2_ in air.

### 3.5. Antimicrobial Assay

**Figure 3:**
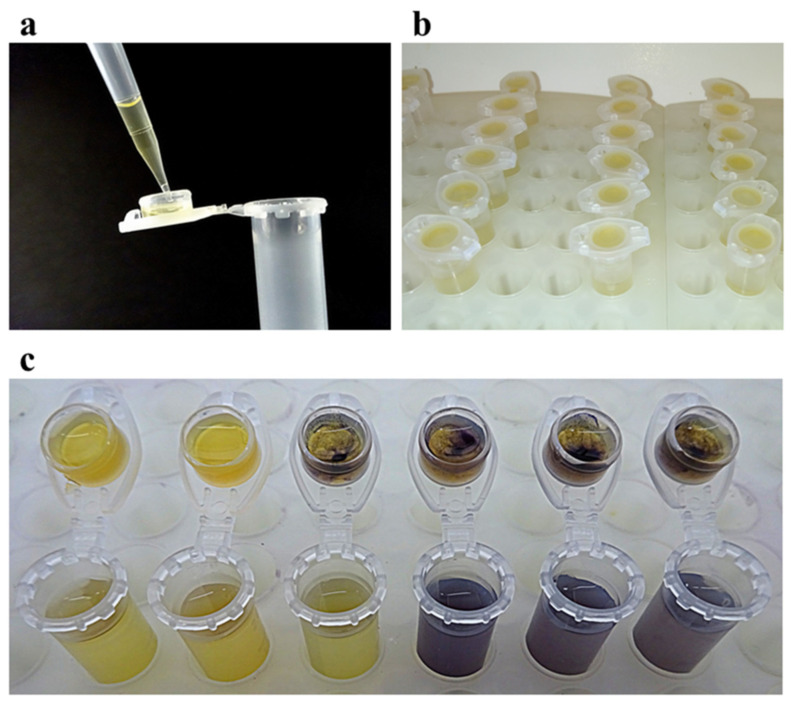
Broth macrodilution volatilization method (**a**) pipetting of agar in the microtube caps: 90 µL of agar is pipetted into rim of every cap; (**b**) incubation: after inoculation, microtubes containing liquid medium with serially diluted samples of tested volatiles and their caps containing solid medium are properly closed together to prevent the losses of active compounds; (**c**) MIC determination: the results are evaluated visually after coloring of living bacterial colonies with MTT dye.

**Figure 4:**
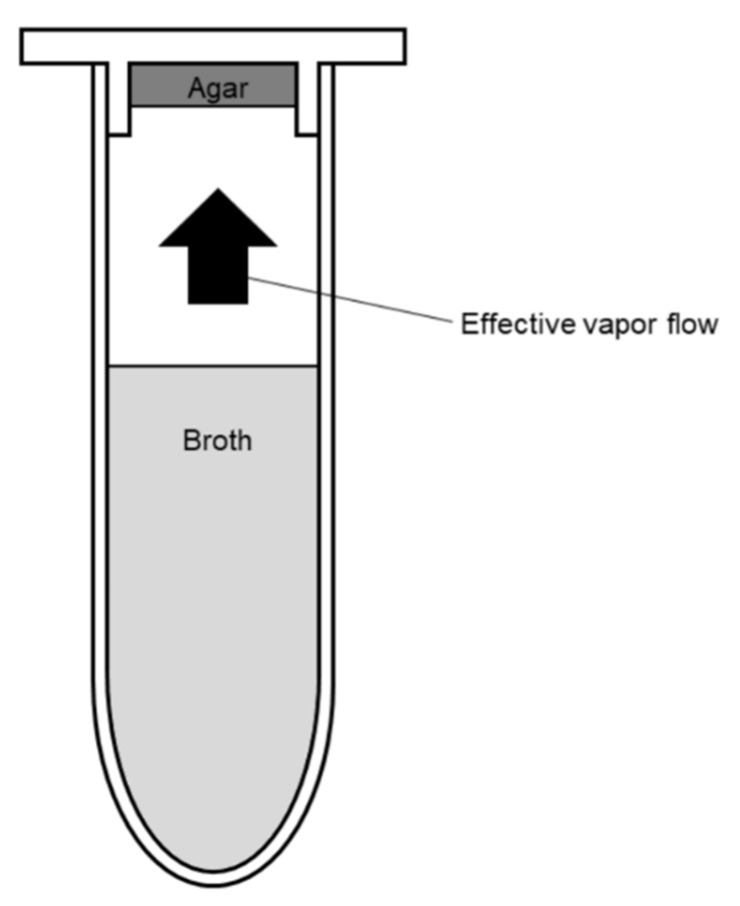
Detail of the cross-sectional view of the closed microtube with snap cap containing broth and agar media.

The antibacterial potential of volatile plant-derived compounds in the liquid and vapor phases was determined using a newly developed broth macrodilution volatilization method performed in standard 2 mL microtubes with snap caps (Eppendorf, Hamburg, Germany). Initially, each sample of compound was dissolved in DMSO at maximum concentration of 1% and diluted in the appropriate broth medium. With the aim to prepare a sufficient amount of stock solutions of the compounds assayed, six two-fold serially diluted concentrations of samples were prepared in 15 mL test tubes closed with plugs to avoid the losses of active compounds by evaporation (Gama Group, Ceske Budejovice, Czech Republic). The concentration of β-thujaplicin started from 1024 µg/mL and from 128 µg/mL for thymohydroquinone and thymoquninone. In the second step, 90 µL of melted agar was pipetted into rims on the caps (Figure 3a) and inoculated with 5 µL of bacterial suspension after agar solidification. Subsequently, the appropriate concentrations of each sample previously prepared in test tubes were pipetted into microtubes in a final volume of 1500 µL. Then, the microtubes were inoculated with 10 µL of bacterial suspension and closed properly (Figure 3b). Microtubes containing inoculated and non-inoculated media were prepared as growth and purity controls simultaneously. After incubation at 37 °C for 24 h, the MICs were evaluated by the visual assessment of bacterial growth after coloring metabolically active bacterial colonies with MTT dye. The respective volumes of 30 and 375 µL of MTT at a concentration of 600 µg/mL were pipetted into the caps and in the microtubes when the interface of color change from yellow to purple (relative to that of colors in control wells) was recorded in broth and agar (Figure 3c). A black and white scheme of a cross-sectional view of a microtube filled with broth and agar shows the effective flow of sample vapors in the closed testing system (Figure 4). The MIC values were determined as the lowest concentrations that inhibited bacterial growth compared with the compound-free control and are expressed in µg/mL. In the case of the vapor phase, the concentration was also expressed in µg/cm^3^ as the weight of the volatile agent per volume unit of a microtube. DMSO, assayed as the negative control, did not inhibit any of the strains at the tested concentration (≤1%). The respective susceptibilities of *H. influenzae*, *S. aureus*, *S. pneumoniae*, and *S. pyogenes* to ampicillin, oxacillin, amoxicillin, and tetracycline were checked as positive antibiotic controls [57]. All tests were performed as three independent experiments, each carried out in triplicate, and the results were presented as median/modal values. According to the widely accepted norm in MIC testing, the mode and median were used for the final value calculation when triplicate endpoints were within the two- and three-dilution ranges, respectively.

### 3.6. Cytotoxicity Assay

A modified method based on the metabolization of MTT to blue formazan by mitochondrial dehydrogenases in living lung cells previously described by Mosmann [58] was used. The lung fibroblast cells were treated for 72 h with the tested compounds dissolved in DMSO at a maximum concentration of 1% and diluted in the EMEM medium supplemented with 10% FBS. Twelve two-fold serially diluted concentrations of these agents ranging from 512 to 0.25 µg/mL were prepared. The microtiter plates were covered with EVA capmats^TM^ at 37 °C in a humidified atmosphere of 5% CO_2_ in air and cultivated for 72 h. Thereafter, MTT reagent (1 mg/mL) in EMEM solution was added to each well and the plates were incubated for an additional 2 h at 37 °C in a humidified atmosphere of 5% CO_2_ in air. The media were removed, and the intracellular formazan product was dissolved in 100 µL of DMSO. The solvent used did not affect the viability of the lung cells at the tested concentration (≤1%). The absorbance was measured at 555 nm using a Tecan Infinite M200 spectrometer (Tecan Group, Mannedorf, Switzerland), and the viability was calculated in comparison to that of the untreated control. Three independent experiments (two replicates each) were performed for each test. The results of the cytotoxicity effect were calculated using GraphPad Prism software (GraphPad Software, La Jolla, CA, USA) and expressed as average IC_50_ with standard deviation in μg/mL. The levels of cytotoxic effects were classified according to the Special Programme for Research and Training in Tropical Diseases (WHO–Tropical Diseases) [46] as cytotoxic (IC_50_ < 2 μg/mL), moderately cytotoxic (IC_50_ 2–89 μg/mL), and non-toxic (IC_50_ > 90 μg/mL). Furthermore, IC_80_ was calculated as equivalent to the MIC endpoint [59] for comparison of microbiological and toxicological data. Therapeutic indices (TIs) were defined as the ratio of $\bar{x}$-IC_80_ and $\bar{x}$-MIC values with the aim of determining the amount of effective antibacterial agents with the quantity causing toxicity [60].

## 4. Conclusions

As a result of this study, a new broth macrodilution volatilization method was developed for the simultaneous determination of the antimicrobial effects of volatile agents in the liquid and the vapor phases at variable concentrations. This rapid, simple, cost- and labor-effective technique, which combines the principles of broth microdilution volatilization and standard broth macrodilution methods, is performed in commercially available microtubes and, therefore, does not require specialized equipment. It can also be a suitable option for the testing of slower growing organisms (e.g., fungi) that require longer incubation time to produce enough growth for MIC determination. Nevertheless, further research focusing on the optimization of the novel broth macrodilution volatilization method for susceptibility testing of a broader spectrum of microorganisms will be necessary to confirm this assumption. In addition, the validity of the method for the susceptibility testing of bacterial pathogens causing respiratory infection was evaluated using three antimicrobial phytochemicals (β-thujaplicin, thymohydroquinone, and thymoquinone). As a result of this research, thymohydroquinone was found to be a promising antibacterial agent for application in inhalation therapy that is safe to human lung cell lines. However, in vivo experiments are required to verify the therapeutic potential of this compound.

## Figures and Tables

**Table 1:** Antibacterial activity of plant volatile compounds in the liquid and vapor phases against respiratory pathogens.

Plant Volatile Compound	Bacterium/Minimal Inhibitory Concentration												$\bar{x}$-MIC
	*Haemophilus influenzae*			*Staphylococcus aureus*			*Streptococcus pneumoniae*			*Streptococcus pyogenes*			
	Broth	Agar		Broth	Agar		Broth	Agar		Broth	Agar		
	(µg/mL)	(µg/mL)	(µg/cm^3^)	(µg/mL)	(µg/mL)	(µg/cm^3^)	(µg/mL)	(µg/mL)	(µg/cm^3^)	(µg/mL)	(µg/mL)	(µg/cm^3^)	
β-thujaplicin	64	512	320	64	512	320	64	1024	640	64	1024	640	64
thymohydroquinone	4	8	5	8	16	10	16	32	20	32	32	20	15
thymoquinone	4	8	5	8	16	10	16	16	10	32	32	20	15
positive antibiotic control	1 ^a^	n.d.	n.d.	0.5 ^b^	n.d.	n.d.	0.25 ^c^	n.d.	n.d.	0.25 ^d^	n.d.	n.d.	-

$\bar{x}$-MIC: mean value of minimal inhibitory concentrations in broth medium, positive antibiotic control: ^a^ ampicillin, ^b^ oxacillin, ^c^ amoxicillin, ^d^ tetracycline, n.d.: not detected.

**Table 2:** Cytotoxicity of plant volatile compounds to the normal lung fibroblast cells MRC-5.

Samples	IC_50_ ± SD (µg/mL)	IC_80_ ± SD (µg/mL)	TI
Plant volatile compound			
β-thujaplicin	4.15 ± 0.45	214.85 ± 9.71	3.36
thymohydroquinone	2.64 ± 0.33	>512.00	>34.13
thymoquinone	1.21 ± 0.24	15.00 ± 4.46	1.00
Positive control			
vinorelbin	0.54 ± 0.26	>10	-

IC_50_: half maximal inhibitory concentration of proliferation in μg/mL, IC_80_: 80% inhibitory concentration of proliferation in μg/mL, SD: standard deviation, TI: therapeutic index (TI = IC_80_/$\bar{x}$-MIC).
